# Progress in 3D Bioprinting Technology for Osteochondral Regeneration

**DOI:** 10.3390/pharmaceutics14081578

**Published:** 2022-07-29

**Authors:** Markel Lafuente-Merchan, Sandra Ruiz-Alonso, Fátima García-Villén, Idoia Gallego, Patricia Gálvez-Martín, Laura Saenz-del-Burgo, Jose Luis Pedraz

**Affiliations:** 1 NanoBioCel Group, Laboratory of Pharmaceutics, School of Pharmacy, University of the Basque Country (UPV/EHU), Paseo de la Universidad 7, 01006 Vitoria-Gasteiz, Spain; markel.lafuentem@ehu.eus (M.L.-M.); sandra.ruiz@ehu.eus (S.R.-A.); fatima.garciav@ehu.eus (F.G.-V.); idoia.gallego@ehu.eus (I.G.); 2 Biomedical Research Networking Center in Bioengineering, Biomaterials and Nanomedicine (CIBER-BBN), Health Institute Carlos III, Paseo de la Universidad 7, 01006 Vitoria-Gasteiz, Spain; 3 Bioaraba, NanoBioCel Research Group, 01009 Vitoria-Gasteiz, Spain; 4 R&D Animal and Human Health, Bioibérica S.A.U., 08029 Barcelona, Spain; pgalvez@bioiberica.com

**Keywords:** 3D bioprinting, osteoarthritis, tissue engineering, regenerative medicine, cartilage, bone

## Abstract

Osteochondral injuries can lead to osteoarthritis (OA). OA is characterized by the progressive degradation of the cartilage tissue together with bone tissue turnover. Consequently, joint pain, inflammation, and stiffness are common, with joint immobility and dysfunction being the most severe symptoms. The increase in the age of the population, along with the increase in risk factors such as obesity, has led OA to the forefront of disabling diseases. In addition, it not only has an increasing prevalence, but is also an economic burden for health systems. Current treatments are focused on relieving pain and inflammation, but they become ineffective as the disease progresses. Therefore, new therapeutic approaches, such as tissue engineering and 3D bioprinting, have emerged. In this review, the advantages of using 3D bioprinting techniques for osteochondral regeneration are described. Furthermore, the biomaterials, cell types, and active molecules that are commonly used for these purposes are indicated. Finally, the most recent promising results for the regeneration of cartilage, bone, and/or the osteochondral unit through 3D bioprinting technologies are considered, as this could be a feasible therapeutic approach to the treatment of OA.

## 1. Introduction

The aging of the population, together with the increase in the prevalence of risk factors such as obesity, physical inactivity, and extreme exercise, has placed osteoarticular diseases in the focus of medicine. Osteochondral defects are characterized by cartilage disruption together with bone damage. Joint traumas and injuries are the most common causes of osteochondral defects [1]. Nevertheless, joint tumors and infections can also be the triggers of osteochondral damage [2,3]. Furthermore, the rare disease osteochondritis dissecans should be also taken into consideration [4]. However, among them, osteoarthritis (OA) has gained notoriety by becoming the third most common condition associated with disability, after dementia and diabetes [5]. In fact, it is estimated that 250 million people are affected worldwide, and that the proportion of the population with an OA diagnosis will increase by 3% by the year 2032 [6]. This increase in prevalence will not only worsen the quality of life of the affected population, but will also entail an economic cost to healthcare systems.

### 1.1. Prevalence and Economic Burden

OA is a disease of the joints such as the knee, hip, and hand that affects 7% of the world population [7]. For example, in the US alone, 37% of people over the age of 65 suffer from this disease [8]. Age is the most important risk factor for the onset of the disease. Likewise, obesity is considered to be another important factor that contributes to the appearance of OA. In fact, due to the aging of the population and the rise in obesity rates, the prevalence of OA has risen by 48% in the last 30 years [9]. Furthermore, it is estimated that the prevalence of OA in the population aged over 45 will increase in the coming years. In addition, it has been shown that hard work activities, high-impact sports, and genetics can also influence the appearance of OA in younger individuals [6].

**Figure 1:**
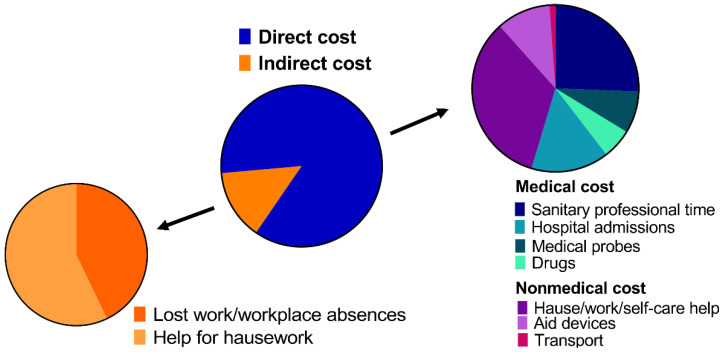
The economic cost of knee and hip OA in Spain. Data from [14].

Given the increase in the disease’s incidence, the economic costs associated with OA must be considered as another problem. In the US, it is estimated that the economic burden ranges between USD 3.4 and 13.2 billion per year [8]. Globally, it is estimated that the medical costs associated with OA in rich countries are between 1 and 2.5% of the gross domestic product [6,10]. However, indirect costs due to work loss, medical leave, and premature retirements could increase the economic burden [11]. In fact, according to the World Health Organization (WHO), OA is in the top 10 diseases that cause work loss due to disability [12]. When it comes to indicating each cost separately, there is considerable disagreement in the literature due to the lack of uniform criteria across the studies, the country where the study was carried out, and the anatomical location and stage of OA [13]. For instance, in Spain, the annual cost is estimated at around EUR 1500 per patient. In addition, Loza et al. [13] conducted a breakdown of the economic expenses associated with knee and hip OA in Spain (Figure 1). According to them, direct costs were around 86%, which could be separated into medical costs (47%) and nonmedical costs (39%). Medical costs included expenses in terms of sanitary professional time (22%), hospital admissions (13%), medical tests and probes (7%), and drug costs (5%). Nonmedical costs were mainly related to house, work, and self-care assistance (29%), aid services (9%), and patient transport costs (1%). On the other hand, indirect costs were estimated to account for 14%; 8% went on assistance for housework, and 6% was due to loss of work, workplace absences, and a decrease in productivity [14]. Nevertheless, the total cost of OA drastically increased when the disease was in the severe stages and when the patients required hospitalization [13].

Due to the increase in cases that not only worsen the quality of life of those affected patients, but also have a great economic cost, improvement of the treatment of this disease is a necessity.

Current treatments are mainly based on palliative drugs, with surgery being the last resort, and only in the most severe cases. However, as these treatments have been shown to be ineffective in the majority of cases, there is a necessity in the scientific community to develop new therapeutic approaches. In this regard, tissue engineering has drawn attention, since it combines different biomedical fields such as cell therapy, nanotechnology, and biomaterial science [15]. Furthermore, additive manufacturing technologies such as 3D bioprinting have emerged to facilitate tissue engineering purposes in a rapid and automatic manner [16]. Thus, the biofabrication of functional scaffolds that could regenerate damaged tissues is currently at its peak. However, prior to the fabrication of artificial tissues, it is necessary to achieve in-depth knowledge of the osteochondral tissue and OA.

### 1.2. Joint Anatomy and Physiology

**Figure 2:**
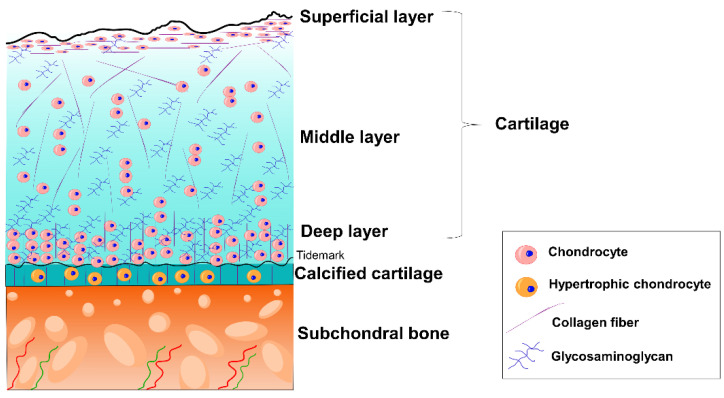
Schematic organization of osteochondral tissue.

Injuries to the osteochondral tissue may lead to OA. Thus, OA is a disorder that affects the whole joint [6]. Joints are areas of articulation between adjacent bones and cartilage for the purpose of providing stability and mobility [17]. Figure 2 shows the schematic organization of the joint tissue separated into two areas: cartilage and bone. At the same time, both cartilage and bone have different layers with their own composition and characteristics.

Cartilage is an avascular, aneural, and alymphatic tissue that is found at the end of long bones [6]. It is composed of highly specialized cells known as chondrocytes and an extracellular matrix (ECM). This ECM is constituted of glycosaminoglycans (GAGs) and collagens that allow the retention of large amounts of water. Considering the avascular nature of the cartilage, this fluid not only allows the chondrocytes to be supplied with nutrients, but is also responsible for providing resistance to mechanical compression [18]. Consequently, cartilage is a tissue with significant biomechanics. For instance, the Young’s modulus of cartilage is between 0.2 and 2 MPa [19]. At the same time, the articular cartilage is morphologically classified into three zones depending on chondrocyte organization, collagen fibril orientation, and GAG content.

The *superficial layer* is the thinner layer, and protects deeper layers from shear stresses. It is characterized by flattered chondrocytes, collagen fibrils oriented parallel to the articular surface, and low GAG content. The *middle layer* is the thickest layer, and functions as an anatomical and functional zone between the superficial layer and the deep layer. Chondrocytes are at low density and spherical, collagen fibrils are obliquely oriented, and the GAG content is increased. This layer is the first line of resistance to compressive forces. Finally, there is the *deep layer*. In this zone, chondrocyte density is increased, and they are arranged in columnar orientation. Collagen fibrils are orientated perpendicular to the surface, and the GAG content is the highest. The deep layer provides the greatest resistance to compressive forces [20,21,22].

Between the bone and the cartilage, there is a zone called *calcified cartilage*. This layer is separated from the deep cartilage layer by a boundary called the *tidemark* that represents the mineralization front [20,23]. Calcified cartilage is composed of hypertrophic chondrocytes, and its main function is to maintain the adhesion of the cartilage to the bone by anchoring the collagen fibrils of the deep zone to the subchondral bone [20,22,23,24].

Bone tissue, known as subchondral bone, is a fundamental tissue for the joint’s proper functionality, since it absorbs the impacts and provides support. In addition, it distributes the mechanical loads throughout the joint with a gradual transition in stress and strain. In fact, bone is considered to be a hard tissue, and its average Young’s modulus ranges from 10 to 20 GPa [25]. This tissue is separated into two zones: *subchondral bone plate* and *subchondral bone trabeculae*. The first layer is a thin cortical lamella lying immediately under the calcified cartilage. It is composed of channels to circulate blood and lymphatic fluid from the bone trabeculae to the cartilage. In contrast, the *subchondral bone trabeculae* are more porous and metabolically active. They contain blood vessels, nerves, and bone marrow that supply the cartilage with nutrients and help in its metabolism [20,23,24].

Apart from osteochondral tissue, the joint unit is also composed of the synovial membrane and synovial fluid, which are involved in the pathogenesis of OA. The synovial membrane is a thin, non-articular layer composed of two cell types: (i) macrophages as a part of the immune system in the joint, and (ii) fibroblasts that secrete synovial fluid. Synovial fluid acts as a lubricant for the articular surface, and transports nutrients to the cartilage [17,20,24].

### 1.3. OA

#### Pathogenesis and Symptomatology

**Figure 3:**
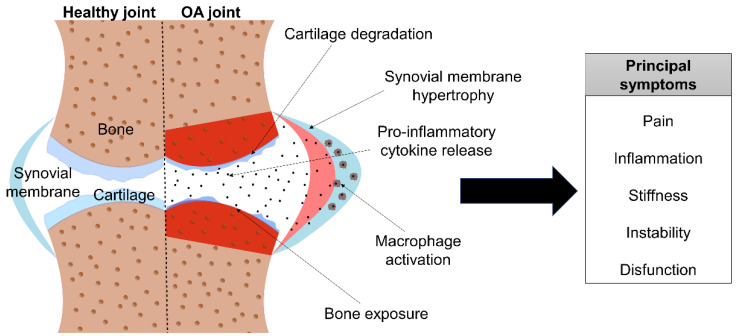
Schematic image of OA’s pathology and symptoms.

In OA, structural alterations in the articular cartilage and subchondral bone are found. As Figure 3 shows, cartilage loses its integrity and, thus, is more exposed to disruption from physical forces. To counteract cartilage erosion, chondrocytes increase the secretion of molecules that cause matrix degradation and pro-inflammatory mediators. On the other hand, bone turnover is increased, developing bone marrow lesions. In addition, there is a vascular invasion throughout the area, and the synovial membrane hypertrophies and macrophages are activated, releasing pro-inflammatory cytokines. Moreover, surrounding tissues, such as ligaments and periarticular muscles, are often affected as well [6]. OA is classified into four different stages, depending on the severity grade and the appearance of the symptoms.

In stage 0, also known as the pre-osteoarthritis stage, the joint seems normal and healthy. Nevertheless, cellular damage starts to occur without any symptoms. Stage 1, or the early stage, is characterized by the appearance of bone spurs, and cartilage begins to lose its integrity. In this stage the patient usually has no symptoms or only mild pain. Then, in the next stage—stage 2, or the mild stage—the cartilage starts to degrade due to enzyme release. Consequently, bone spurs grow and become painful. Joint pain and stiffness commonly appear during activities at this stage. As the disease progresses, stage 3 or the moderate stage appears. Here, cartilage shows obvious damage, and the space between the joints becomes narrower. Therefore, pain while moving is frequent, and joint stiffness worsens. Finally, stage 4, or severe OA, occurs. This stage is characterized by the presence of little cartilage—or the absence of cartilage in severe cases. Synovial fluid is reduced, and bone may erode, provoking bone narrow damage. At this stage, significant pain and discomfort appear, stiffness and inflammation are severe, and joint dysfunction may occur [26,27,28].

In summary, affected people experience pain and inflammation, which are the most common and disabling symptoms. Moreover, muscle weakness and joint instability are frequent symptoms. Apart from physical symptomatology, psychological disorders due to pain together with insomnia and fatigue should be taken into account [6,9].

## 2. Current Treatments

OA is a progressive and degenerative joint disease with no cure. Treatments in the early stages of OA are focused on giving educational information to patients, weight loss, and moderate physical exercise, whereas when the disorder progresses, current treatment is based on alleviating the main disabling symptom—the chronic pain [29]. Therapeutic guidelines recommend starting with topical treatment and moving on to oral treatment when topical drugs do not relieve the pain. In more advanced stages of OA, intra-articular injections are the recommended treatment. Finally, when OA is in the severe stages and the aforementioned treatment becomes ineffective, surgery may be recommended. All of these treatments are summarized in Table 1.

### 2.1. Pharmacological Treatments

Topical treatment is based on topical nonsteroidal anti-inflammatory drugs (NSAIDs). These demonstrate good effectiveness for pain reduction, and the side effects that they may produce are rare. As they are usually well-tolerated and have an easy mode of administration, they are highly recommended in the first stages of the disease. Nevertheless, they become ineffective as the disease progresses and the pain increases [6,30,31].

When pain increases, oral drug intake is recommended. Among the used drugs, acetaminophen or paracetamol is a well-known drug to reduce mild-to-moderate pain [6,30]. It is usually prescribed because several guidelines recommend it for OA. However, there is an increasing controversy about its efficacy in this illness. Meta-analyses have revealed little in the way of satisfactory effects in comparison with placebo [6,30]. Furthermore, the hepatotoxic side effects after long-term usage with high doses are a drawback to take into account [30]. Therefore, the use of this drug may be restricted to short-term periods [31].

NSAIDs are the treatment of choice, since it has been shown that they decrease pain and improve joint function [30]. However, long-term treatments at high doses have considerable side effects, such as gastrointestinal issues and nephrotoxicity [30,31]. COX-2-selective inhibitors are a form of NSAIDs that may avoid these problems, but are contraindicated in patients with cardiovascular problems [31]. As a consequence, their use should be restricted to short treatment periods, making a good therapeutic approach for chronic diseases such as OA impossible.

Symptomatic slow-acting drugs in OA (SYSADOA), such as glucosamine and chondroitin sulfate (CS), are widely prescribed. CS is a GAG naturally found in the ECM, whereas glucosamine is a metabolic precursor of GAGs. There is much controversy in the literature regarding the use of these substances. They have the advantage of being safe and showing almost no side effects, but their therapeutic mechanism is unclear, and while some meta-analyses indicate their potential benefits in pain relief and improvement in physical joint function, others strongly discourage their use [30,31].

For patients who do not respond well to oral treatments, intra-articular injections of hyaluronic acid (HA) are recommended. HA is a molecule from the group of GAGs that are naturally found in the joints’ synovial fluid. Its main function is the lubrication of the joints. It has also been reported to be chondroprotective against mechanical damage. It has been found that intra-articular injections of HA reduce pain, have anti-inflammatory effects, and promote GAG synthesis. It is generally safe and effective in mild-to-moderate stages of knee OA. As a drawback, its long-term effects are limited, and repetitive injections of HA are usually uncomfortable for patients [30,31].

In addition, intra-articular corticosteroids are very common drugs used to treat inflammatory-related diseases; therefore, they are expected to be beneficial to treat OA as well, by reducing joint inflammation, pain, and dysfunction. All of the clinical evidence has demonstrated pain reduction after corticosteroid injections. Nevertheless, this benefit has only been observed in short-term periods, and repetitive injections have not been associated with long-term pain reduction. In addition, as the studies have focused on knee joint treatment, whether these drugs are beneficial for other joints is unclear. Furthermore, a recent meta-analysis showed little improvement in joint function, and reported greater cartilage damage than in the placebo group after 2 years of corticosteroid administration. Thus, the use of this kind of drug for OA treatment has become controversial [6,30].

As an alternative, some guidelines recommend the use of opiates. Although they are quite effective in relieving pain, the side effects that they produce are extensive and serious, such as tolerance and dependence. In fact, opioid abuse has been recognized as an epidemic in the US, where great efforts are being made in order to reduce their use [32]. Apart from this serious problem, opioids have shown only a small improvement in the OA symptomatology, with an increase in side effects after opiate administration. Thus, the use of opiates is highly discouraged, and they should only be prescribed for short-term treatments and when other therapeutic options are not possible [6,30,31].

### 2.2. Surgery

Different surgical interventions have been carried out in the clinics. A bone marrow stimulation technique known as microfracture is recommended to treat small chondral defects (less than 2 cm^2^). When the disease reaches the subchondral bone, osteochondral autograft transplantation is the suggested option. This surgical procedure is divided into mosaicplasty, which is based on transplanting multiple small, circular osteochondral grafts, and the single-plug technique, which consists of implanting a single, larger graft. Excellent results have been reported after the implementation of both techniques, but there are still disadvantages in terms of donor site morbidity and patient age limitation, as the procedure is restricted to patients over 50 years old [33]. In order to treat greater osteochondral defects, allograft implantation has been suggested, but there may be limitations in acquiring the graft due to donor unavailability [34].

Total joint replacement surgery or arthroplasty is the last therapeutic option. During this surgery, the damaged joint is replaced with an artificial implant that is made of metal, ceramic, or plastic [33]. This is recommended for patients with severe OA whose quality of life is considerably reduced. Clinically relevant improvements have been observed, but complications associated with surgery in the elderly population are common. Infections, neurovascular injury, and peri-implant fractures are the main complications. Additionally, implant rejections are around 12%, and pain is still a recurrent symptom [6,30].

### 2.3. Biological Therapies

Recently, apart from traditional drugs and surgery, treatments based on intra-articular injection of platelet-rich plasma (PRP) have attracted significant clinical interest. This therapy consists of inoculating autologous plasma on the joint, because it has been shown that the PRP releases bioactive molecules (e.g., growth factors, cytokines, or anti-inflammatory mediators). This has been reported to relieve OA symptoms and to demonstrate no side effects. Nevertheless, PRP is limited to the knee joint, and variability has been observed among patients. This can be explained by the absence of a clear dosage guide and the lack of a standardized plasma extraction protocol. Moreover, simultaneous treatment with PRP and NSAIDs reduces the PRP’s efficacy [35,36].

Autologous chondrocyte implantation (ACI) has also been used for the treatment of osteochondral defects for years. This treatment, authorized by the European Medicines Agency (EMA) as an advanced therapy medicinal product, is based on the implantation of autologous chondrocytes in the joint to promote its regeneration and, therefore, alleviate the symptoms. In fact, Spherox™ is the only ACI product that has been commercialized. This therapy is based on the implantation of spherical aggregates that are composed of autologous human chondrocytes expanded ex vivo and an auto-synthetized extracellular matrix. However, this treatment has also shown some drawbacks, such as the necessity of expert surgeons for its application, as well as the authorization of the hospital in which the therapy is applied. Moreover, ex vivo cell expansion requires strong regulatory procedures. Consequently, this treatment is not available in all hospitals, and it has high costs. In addition, it is contraindicated in advanced OA stages (i.e., stages 3 and 4), and it is only prescribed for knee joint defects [37].

In conclusion, current treatments can alleviate the symptoms produced by OA in the short-term. In the long-term, as the disease progresses and the pain becomes intense, current treatments fail to improve the patient’s quality of life. In addition, given that the target population that suffers from OA is the elderly, the appearance of other diseases that could hinder the general use of these drugs should be highlighted. Therefore, new therapeutic approaches should be proposed.

## 3. New Therapeutic Approaches: Tissue Engineering and 3D Bioprinting

As mentioned above, one of the most interesting therapeutic approaches for OA is the use of ACI. This kind of cell therapy has been widely researched and improved, since it can protect cartilage from degradation and, consequently, cause remission of the disease’s symptomatology. In fact, there are several studies, including clinical trials, in which this therapy has shown promising results [37]. Another approach that is gaining attention in cell therapy treatments is the use of mesenchymal stromal cells (MSCs), as it has been reported that the articular administration of MSCs in the knee relieves pain and improves its function [38]. Likewise, adipose-derived stromal cells (ASCs) have also been applied for chondral regeneration, since they can be harvested with reduced mobility at the donor site in comparison with other MSC sources [39]. Furthermore, both ASCs and MSCs have the potential to secrete anti-inflammatory and immunomodulatory molecules, which complement their administration as an OA treatment [40,41]. However, the long-term benefits are controversial. In addition, it has been proven that injection of MSCs through a needle compromises their viability due to shear forces and that, after administration, cells tend to migrate, making it difficult to secrete therapeutically active molecules [40]. The implementation of a cellular support would not only avoid these drawbacks, but also take into account the mechanical properties that are of great importance in the regeneration of the joint. In fact, hydrogel-based cellular supports have been already studied with successful results in terms of mimicking native mechanical properties and improving cell viability [42,43].

**Figure 4:**
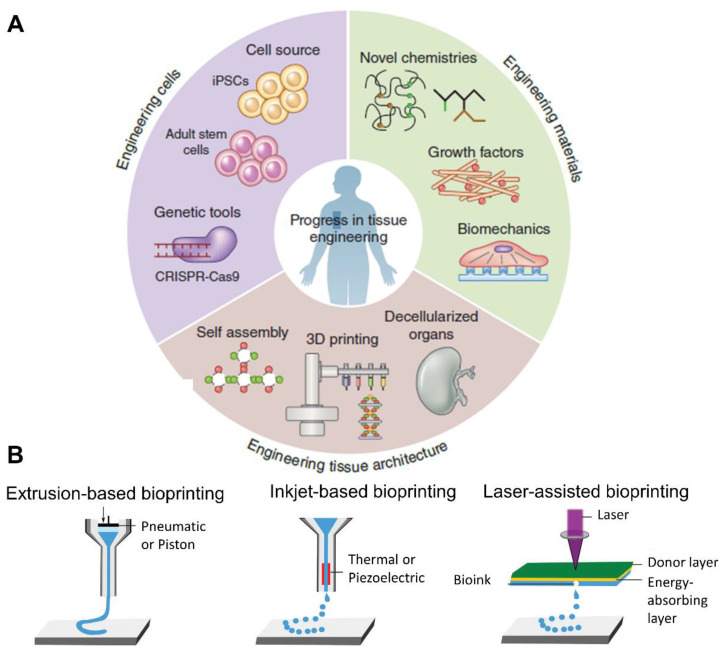
(**A**) Diagram of the elements used in tissue engineering. Adapted from [46]. (**B**) Scheme of different bioprinting methods. Adapted from [47].

In this context, tissue engineering, which brings together cell therapy, biomaterial engineering, and the delivery of drugs or therapeutic molecules, has become the most promising therapeutic approach (Figure 4) [16,44,45]. It is based on the manufacture of three-dimensional (3D) structures or scaffolds that support cells, allowing them to adhere, proliferate, and differentiate. These structures can also contain different elements, such as drugs, growth factors, and therapeutic molecules [16].

Among scaffold manufacturing techniques, 3D bioprinting has gained significance in recent years. This additive manufacturing technique is characterized by the fabrication of layer-by-layer structures via computer-aided design (CAD). CAD generates a G-code that can be read by the bioprinter [48]. The creation of the design in CAD as well as the modification of the G-code allows the total control of the shape and structure of the scaffold, granting bioprinting an advantage over traditional manufacturing techniques. Furthermore, bioprinting techniques permit the addition of high cell densities, while other techniques are unable to do so, or the cells have to be added after making the scaffold [49]. In fact, anatomically specific implants could be designed for each patient using this technology. Another challenge in the tissue engineering field for joint regeneration is the fact that the joint is made up of two tissues—cartilage and bone—which, in turn, have separate zones with different cell densities, compositions, and biomechanics. Three-dimensional bioprinting, as an additive technique, allows the manufacturing of scaffolds with different layers; therefore, the native tissue can be imitated. In addition, it is fast and automatic, and accepts a wide variety of materials, wmaking it a promising technique in this field [48,50].

The deposited material is known as bio-ink. These bio-inks are composed of cells and biomaterials to which other molecules such as drugs, proteins, genetic material, or growth factors may be added [51]. However, the biomaterial, whether of natural or synthetic origin, has to meet certain requirements to be considered a bio-ink [50]. In the first place, it has to be biocompatible with the cells, since it has to support cell attachment, migration, proliferation, and differentiation. Second, it has to be biodegradable and, finally, it has to be printable, which necessitates taking into account its rheological properties and gelation kinetics. Moreover, the biomaterial must have proper mechanical properties and be bioactive [52].

There are different 3D bioprinting techniques, including extrusion-based, inkjet-based, and laser-assisted bioprinting (Figure 4B). Each of these techniques is based on different principles. *Extrusion-based bioprinting* is the most common technique, since it is easy to use, economical, and flexible in the use of a wide range of materials [53]. It is based on the continuous deposition of the bio-ink in a filament form through a needle via the application of mechanical pressure or air pressure (pneumatic). *Inkjet-based bioprinting* is characterized by the deposition of the bio-ink in a droplet form after the application of a piezoelectric or electrostatic drop-on-demand source. *Laser-assisted bioprinting* uses a laser energy beam for the deposition of the bio-ink [53,54].

Several studies have implemented bioprinting techniques for joint regeneration. The majority of those studies focus on one of the two tissues involved—cartilage or bone—whereas fewer studies are based on the development of the whole osteochondral unit by 3D bioprinting.

### 3.1. 3D Bioprinting in Cartilage

Cartilage tissue lacks blood vessels, nerves, and a lymphatic system, making this tissue an ideal target for 3D bioprinting in comparison with other, more complex tissues [24,44]. Nevertheless, cartilage is subjected to high shear forces. Thus, the challenge, when it comes to bioprinting cartilage, is to meet the requirements in terms of mechanical properties, as well as to mimic the layered structure of the native tissue as closely as possible.

To obtain 3D structures resistant to mechanical pressures, some studies have focused on seeding cells on previously 3D-printed scaffolds, using thermoplastic polymers such as polycaprolactone (PCL) or polylactic acid (PLA) [55]. Electrospinning, which enables the fabrication of polymeric fibers, is another innovative technology that has been used for this purpose, since nanofibers reinforce the scaffold [56]. However, 3D bioprinting technology requires the inclusion of the biological part (living cells) in the bio-ink, so the use of these materials is unsuitable for cells, as these polymers need high temperatures to be extruded. For this reason, hydrogels are the most used option, as they have the ability to absorb water, are biocompatible with the cells, and are biodegradable.

Different biomaterials have been studied to develop bio-inks. Among them, biomaterials that are naturally found in osteochondral tissue—such as collagens or GAGs—have been proposed, as along with others that have greater printability characteristics or mechanical resistance, such as alginate, gelatin, or silk fibroin. All of these studies are represented in Table 2.

**Figure 5:**
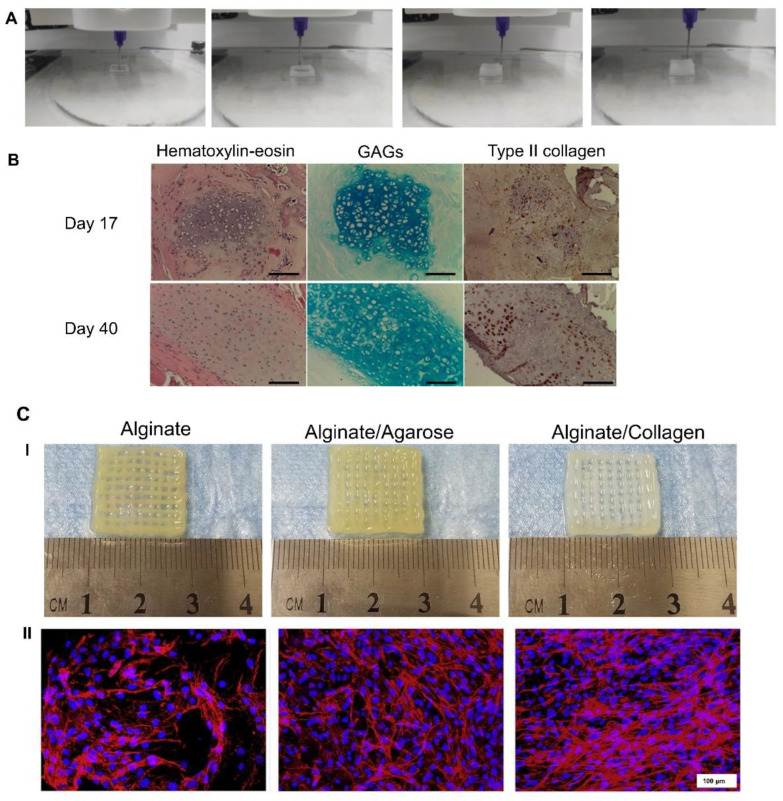
Collagen-based scaffolds: (**A**). Extrusion-based bioprinting of a 4% collagen scaffold. (**B**). Cartilage ECM evaluation after in vivo implantation. At day 40, GAG accumulation and type II collagen production were increased. Scale bar = 100 µm. Adapted from [57]. (**C(I)**). Macroscopic images of alginate, alginate–agarose, and alginate–collagen scaffolds. (**C(II)**). Rhodamine–phalloidin/Hoechst 33,258 staining after 14 days of bioprinting. Scale bar = 100 µm. Adapted from [58].

Collagen has been used for the production of bio-inks because it is an element that is widely distributed among mammalian tissues. Several types of collagen are known, from type I to XXI. However, most studies have focused on type II collagen, which is the main component of the cartilage ECM, and type I collagen, which is abundant in bone tissue. Thus, in one of the studies in which collagen is used, Beketov et al. [57] argued that one of the drawbacks of using collagen is that scaffolds are often quite fragile. Hence, they proposed the use of high concentrations (4%) of type I collagen to develop a bio-ink with embedded rat chondrocytes. Using extrusion-based bioprinting (Figure 5A), they managed to obtain scaffolds with better printability and mechanics than other studies based on scaffolds with lower concentrations of collagen. Interestingly, after an in vivo study in rats, they showed the ability of these scaffolds to form cartilage ECM, rich in type II collagen and GAGs, as shown in Figure 5B [57]. Another proposal to increase the scaffolds’ mechanics is the combination of collagen with another polymer, such as alginate or agarose. Thus, Yang et al. [58] developed alginate-based scaffolds with added collagen and agarose using extrusion-based bioprinting (Figure 5C(I)). They showed that by adding collagen to the scaffold, its mechanical properties, along with the viability of rat primary chondrocytes, increased compared to scaffolds with agarose (Figure 5C(II)). Furthermore, cells inside collagen-containing scaffolds had increased chondrogenic-phenotype gene expression as well as GAG production [58].

**Figure 6:**
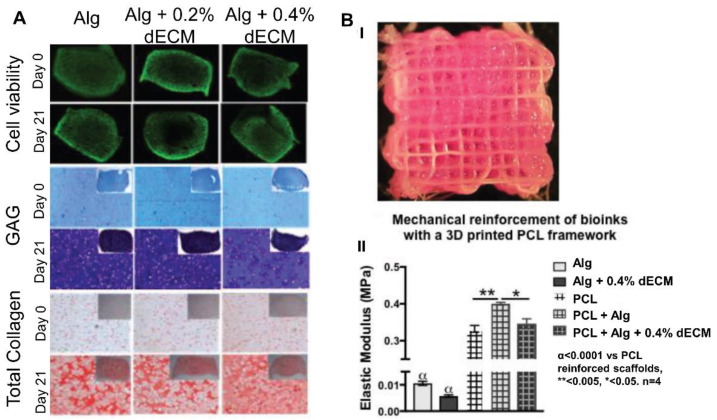
Alginate/dECM-based scaffolds: (**A**). Cell viability, histology, and immunostaining on days 0 and 21 showed good cell viability and high GAG and collagen production within 21 days. (**B**) Alginate/dECM 3D bioprinting and PCL 3D printing combination. (**I**) Representative image of the hybrid scaffold. (**II**) Mechanical properties are enhanced with PCL reinforcement. Adapted from [60].

Alginate (Alg) is a natural polymer that has been widely used for cartilage bioprinting due to its biocompatibility and easy post-bioprinting crosslinking procedure. Nguyen et al. [59] used it as a component of their bio-ink in combination with nanofibrillated cellulose (NFC). On the other hand, they developed bio-inks based on NFC/HA. They embedded bio-inkhuman-derived induced pluripotent stem cells (iPSCs) co-cultured with irradiated human chondrocytes in these two bio-inks, and they bioprinted scaffolds using extrusion-based bioprinting. The scaffolds containing alginate showed better results, as cells maintained their pluripotency and chondrogenic phenotype in comparison with HA scaffolds, in which cells showed low proliferation capacity [59]. Mechanical properties that are of key importance for cartilage were not measured in this research. In another study, Rathan et al. [60] mixed alginate with different concentrations of decellularized pig cartilage ECM (dECM) to obtain ECM-functionalized alginate bio-inks, in which human bone marrow MSCs and the chondrogenic growth factor TFG-β3 were included. They achieved bioprinted scaffolds through extrusion, and a sustained release of the growth factor. Moreover, they demonstrated that by increasing the concentration in the bio-ink from 0.2% to 0.4%bio-ink, cell proliferation and the chondrogenic differentiation were enhanced (Figure 6A). However, the authors suggested that, in a long-term, the osteogenic differentiation could also occur. Finally, they combined 3D bioprinting with 3D printing techniques to include PCL fibers that reinforced the scaffold, thus achieving mechanical properties similar to those of the native cartilage (Figure 6B) [60].

As mentioned above, dECMs have been used as components in the tissue engineering field, as they are biologically and functionally closer to native tissues than polymers [62]. However, the drawback of these components is that they fail to meet the necessary rheological properties to be considered as bio-inks by themselves. Thus, Visscher et al. [61] used gelatin, HA, glycerol, and Dulbecco′s modified Eagle′s medium (DMEM) to fabricate a bio-ink containing pig-cartilage-derived dECM that had been methacrylated. They embedded rabbit chondrocytes into the bio-ink and manufactured scaffolds through extrusion-based bioprinting. Cell viability and proliferation were increased proportionally to the dECM concentration. dECM also promoted cells to produce GAGs and collagen. In addition, the scaffold mechanical properties were also positively improved by the inclusion of dECM [61]. These promising results after including dECM have also been reported by other researchers. Zhang et al. [62] proposed mixing decellularized goat cartilage ECM with silk fibroins. They included rabbit bone marrow MSCs, TGF-β3 as a chondrogenic growth factor, and polyethylene glycol (PEG) 400 as a crosslinker. After a rheological study to determine the optimal concentrations for the bio-ink, they managed to manufacture porous scaffolds via extrusion. The cell viability, proliferation, and chondrogenic differentiation were good, and were proportional to the amount of dECM. Furthermore, they obtained a sustained release of TGF-β3 from the scaffold, promoting the production of collagen and GAGs. Finally, bioprinted scaffolds were implanted subcutaneously in mice. In vivo results showed an increase in the production of GAGs and collagen, as well as in the scaffolds’ mechanical properties, making these scaffolds a promising therapeutic approach for cartilage regeneration [62].

**Figure 7:**
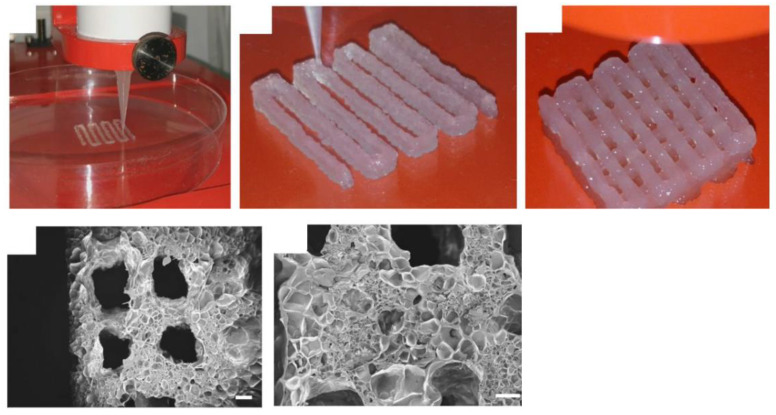
Silk fibroin (SF)-based scaffolds. Extrusion bioprinting process of SF + gelatin bio-ink, obtaining porous scaffolds. Scale bar = 200 µm. Adapted from [64].

In a previous work by the same research group, Li et al. [63] developed a bio-ink by combining silk fibroin, rabbit platelet-rich plasma (PRP), rabbit chondrocytes, and PEG 400. After extrusion bioprinting, they showed that the addition of plasma increased the scaffolds’ mechanical properties as well as cell viability and proliferation. Interestingly, the plasma contained various growth factors that were delivered from the scaffold to promote cell functionality and chondrogenic differentiation [63]. In fact, silk fibroin has gained popularity as a bio-ink component because it is biocompatible, biodegradable, and has remarkable mechanical strength [62]. Another work using silk fibroin as a bio-ink component was the one proposed by Singh et al [64]., who developed a bio-ink based on two types of silk fibroin, gelatin, and porcine primary chondrocytes. They obtained porous and printable scaffolds via extrusion-based bioprinting (Figure 7). Cells showed high viability and proliferation ability inside the scaffolds, as well as chondrogenic gene expression and cartilage ECM production. Moreover, the authors injected the bio-ink subcutaneously in mice to study their immune response. As a result, a long immune response was not found, so they suggested the use of these scaffolds for cartilage regeneration. Nevertheless, the scaffold mechanical properties were lower (143 kPa) than those of native cartilage; therefore, the scaffolds would only be beneficial for soft tissue regeneration [64].

Achieving mechanical properties in the scaffolds that are similar to those of human cartilage is challenging when using hydrogels such as alginate, silk fibroin, or collagen by themselves. Consequently, other bio-ink components have been proposed. For example, Li et al. [65] developed a bio-ink containing chemically modified chitosan to make it soluble, along with chondroitin sulfate, since it has been reported to be involved in the mechanical response of native cartilage as well as in cartilage regeneration [73]. They included human adipose-derived MSCs and used pluronic as sacrificial ink to give support to the scaffold. Once they manufactured the scaffolds by extrusion, they studied their biocompatibility in mice by subcutaneous implantation. Immune response decreased within the following days after implantation. Interestingly, cartilage-degradative cytokines were reduced as a consequence of the chondroitin’s anti-inflammatory effects, suggesting the use of chondroitin for cartilage regeneration purposes [65]. However, the scaffolds’ mechanical properties and how the chondroitin was involved in them were not shown.

**Figure 8:**
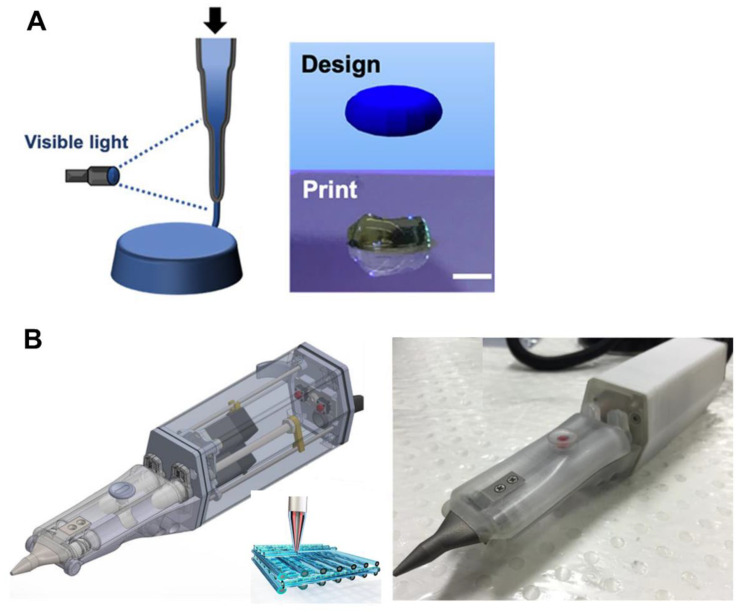
In situ bioprinting techniques: (**A**) In situ crosslinking technique consisting of exposing the bio-ink to visible light just after being extruded. Adapted from [66]. (**B**) “Biopen”—extrusion-based handled bioprinting technique based on a coaxial system. Adapted from [67].

Similar to chondroitin sulfate, HA has been reported to have promising properties for regenerating cartilage. However, it has poor rheological properties for use as a bio-ink without any other supportive component(s). Galarraga et al. [66] modified hyaluronic acid to create norbornene-modified HA, which was crosslinkable with visible light. In addition, they developed an in situ crosslinking technique that consisted of exposing the bio-ink to visible light just after being extruded (Figure 8A). Thus, they developed HA scaffolds without the addition of any rheological component to the bio-ink. Bovine bone marrow MSCs were viable after bioprinting, indicating that the technique was biocompatible. Moreover, cells’ chondrogenic differentiation and scaffold mechanical properties increased after bioprinting [66]. Other research groups have also developed in situ bioprinting techniques. In the case of Di Bella et al [67]., they used an extrusion-based handled bioprinting technique based on a coaxial system called “Biopen” (Figure 8A). The bio-ink was made of ovine adipose-derived MSCs, hyaluronic methacrylate, and gelatin methacrylate. The authors demonstrated high cell viability after using this technique in a previous work [74], and this study was focused on in vivo research using a chondral defect sheep model. They compared the use of the Biopen with scaffolds bioprinted using a conventional bioprinter. As a result, no differences were observed in terms of cartilage regeneration and mechanical properties between the scaffolds created by the Biopen and the conventional bioprinter; in fact, they were good in both cases. Nevertheless, Biopen-fabricated scaffolds showed better overall macroscopic and microscopic characteristics, together with excellent applicability and handling of the technique by the surgeons. As a point for improvement, both implants failed to adhere to the host tissue. Therefore, chemical modifications of the bio-ink could be needed [67].

**Figure 9:**
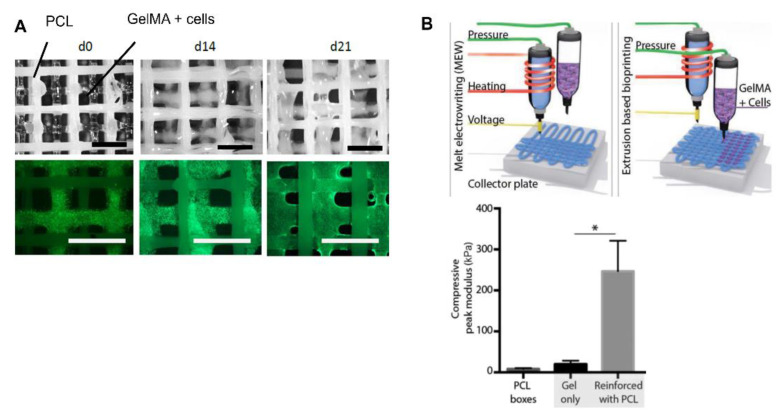
GelMA-based scaffold: (**A**) Representative bright-field and fluorescence images of hybrid scaffolds composed of PCL and GelMA. Scale bar = 2 mm. Adapted from [68]. (**B**) Schematic image of extrusion-based bioprinting and electrowriting techniques that improved scaffold mechanical properties * = *p* < 0.05. Adapted from [69].

Gelatin methacrylate (GelMA) has also been widely applied in cartilage regeneration via 3D bioprinting due to its desirable fast crosslinking using UV light, together with its biodegradability, biocompatibility, and limited antigenicity. Ruiz-Cantu et al. [68] used extrusion-based bioprinting to manufacture porous scaffolds using a GelMA bio-ink with ovine chondrocytes. GelMA, as a cell carrier, proved to be good in terms of high cell viability and proliferation after bioprinting. In addition, cells also managed to produce GAGs and collagen. Despite the fact that mechanical properties increased after bioprinting, they did not achieve native cartilage values. Consequently, the authors proposed the addition of PCL through 3D printing as a mechanical support. The hybrid GelMA–PCL scaffold showed the same ability to maintain good cell viability (Figure 9A) and chondrogenic functionality after bioprinting. Interestingly, the mechanical properties strongly increased, suggesting the use of hybrid scaffolds as a cartilage regeneration strategy [68]. A similar approach was performed by de Ruijter et al. [69] In this case, the scaffold bioprinted by extrusion, and composed of GelMA and equine MSCs, was reinforced with PCL fibers using a melt electrowriting technique. This electrospinning technology uses a high-voltage electrical field to form sub-micrometer fibers from polymer melts. The authors showed that the inclusion of this technique had no negative effects on cell viability and proliferation. In fact, cells maintained the ability to produce GAGs and collagen after bioprinting. Therefore, as Figure 9B shows, the inclusion of PCL with this technique may be a good option to increase the mechanical properties of scaffolds for cartilage regeneration [69].

Zhu et al. [70] proposed another bioprinting approach using GelMA/human bone marrow MSCs bio-ink. First, they included polyethylene glycol diacrylate (PEGDA) in order to increase the mechanical properties. Then, they added the growth factor TFG-β1 encapsulated in poly-lactic-co-glycolic acid (PLGA) to promote chondrogenic differentiation. Finally, they used stereolithography-based 3D bioprinting, which is a laser-assisted bioprinting method to manufacture scaffolds. As a result, they showed that PEGDA improved mechanical properties and printability. Importantly, cell viability and proliferation were high despite the crosslinking procedure with UV light. Moreover, a sustained release of TFG-β1 from the scaffold was found, which enhanced cells’ chondrogenic phenotype expression after bioprinting [70]. Consequently, similar to extrusion-based bioprinting, with this technique, it is possible to obtain adequate scaffolds for cartilage regeneration purposes.

**Figure 10:**
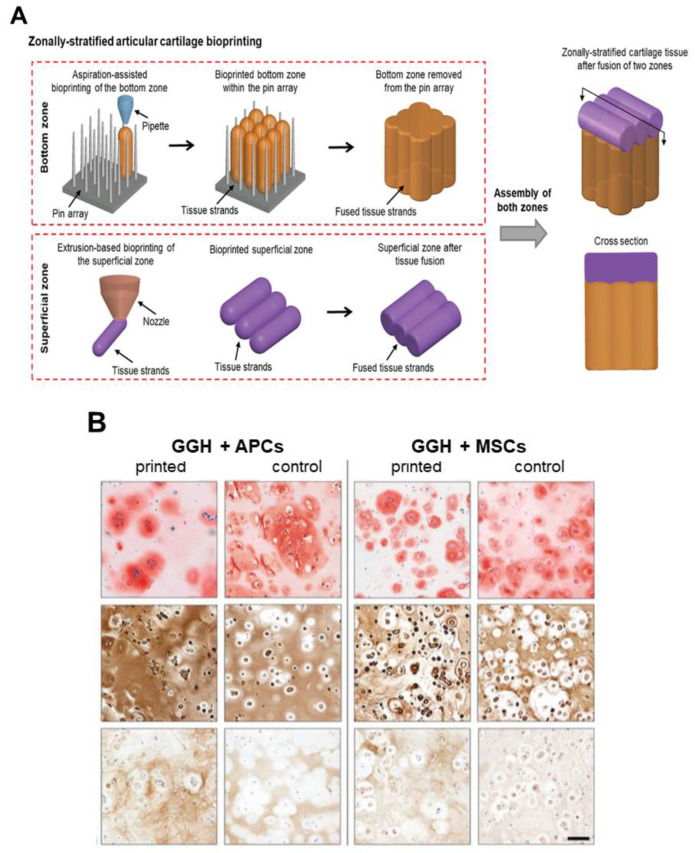
Layered scaffolds: (**A**) Schematic image of the manufacture of zonally stratified articular cartilage. Adapted from [71]. (**B**). Histological images of GAGs (safranin-O, **top**), collagen type II (**middle**), and collagen type I (**bottom**) matrix of APCs and MSCs in GelMA/gellan gum/HAMA (GGH) bioprinted scaffolds at day 42. Scale bar = 100 μm. Adapted from [72].

Apart from mechanics, the other challenge when it comes to bioprinting structures in order to substitute damaged cartilage is the creation of scaffolds that simulate the internal layered structure of the native cartilage. To do so, Wu et al. [71] combined extrusion bioprinting with aspiration-assisted bioprinting (AAB), which allows precise positioning of spheroids by employing aspiration to lift individual spheroids and bioprint them onto a hydrogel. By using sodium alginate and human adipose-derived stem cells (ADSCs) they manufactured a two-layered scaffold simulating the deep and superficial layers of the cartilage. As Figure 10A shows, first, they developed the deeper layer through AAB by depositing spheroids vertically with the support of a pin ray. Then, the superficial layer was extruded horizontally on the other layer. The resulting scaffold showed high cell viability after both bioprinting techniques, as well as mechanical properties similar to those of the native cartilage (2.1 MPa). Interestingly, cells deposited collagen fibers aligned similarly to native cartilage [71].

In another recent work, Mouser et al. [72] aimed to develop heterocellular cartilage constructs by using three different cell types: equine chondrocytes, MSCs, and articular cartilage progenitor cells (ACPC) which were reported to be in the superficial layer of the cartilage. To achieve this, cells were embedded into two inks composed of GelMA/gellan gum and GelMA/gellan gun/hyaluronic methacrylate (HAMA). Then, the authors created scaffolds using extrusion-based bioprinting. First, after evaluating which bio-ink had better results, they concluded that the addition of HAMA considerably improved the printability. Among the cells, the non-differentiated ones showed higher cartilage ECM production, but there were no significant differences in terms of including them in one ink or the other. Taking into account these data, they used GelMA/gellan gum/HAMA (GGH) bio-ink to fabricate scaffolds with a middle/deep layer containing MSCs and a superficial layer with ACPCs. As a result, the layered scaffold demonstrated positive staining of GAGs and collagen as well as chondrogenic gene expression (Figure 10B). However, the mechanical properties were not specified, and there were no differences between the two different layers; therefore, further studies need to be conducted [72].

### 3.2. 3D Bioprinting in Bone

OA is characterized by osteochondral damage that affects cartilage and bone tissues. For this reason, studies focused on the development of scaffolds using 3D bioprinting technology for bone regeneration have also been carried out. The ideal scaffold should take into account the structure and composition of human bone, and should not only have excellent mechanical properties, but also contain a porous structure, and be both osteoinductive and osteoconductive. In addition, bone tissue contains a vascular system; therefore, scaffolds should provide vascularization to nourish bone cells as well as cartilage tissue.

As a hard tissue, the mechanics and stiffness of the substitute scaffold are of key importance. Consequently, many of the studies use 3D printing technology with synthetic materials in which cells are seeded later on top of the fabricated scaffold [75]. Among them, PCL has gained notoriety due to its good mechanical properties, and because it favors cell adhesion and proliferation [76]. In the field of 3D bioprinting, the use of hydrogels based on polymers such as alginate and GelMA is widespread, since they are good carriers for the cells. However, they have shown low bioactivity, and the bioprinted structures are usually soft and very different from native bone’s mechanics. In order to overcome these inconveniences, researchers have been forced to include other elements, such as ceramics, glasses, or inorganic components (Table 3).

Hydroxyapatite (HAP), as a major inorganic component of bone, has been found to be bioactive and osteoinductive [85]. For this reason, it was included in the work published by Bendtsen et al. [77], who developed alginate/polyvinyl alcohol (PVA)/HAP inks to obtain scaffolds through extrusion-based bioprinting. The addition of PVA/HAP enhanced the rheological properties as well as the viability of murine calvaria 3T3-E1 cells. Importantly, although mechanical properties increased with PVA/HAP, they did not resemble those of bone [77]. Similarly, Keriquel et al. [78] evaluated bioprinted scaffolds for bone regeneration in rat calvaria defects. To do so, a nanohydroxyapatite/collagen type 1 bio-ink with murine D1-MSCs was developed, and scaffolds with two geometries (ring and disk) were manufactured using laser-based bioprinting. Results showed high cell viability and proliferation as well as bone regeneration and formation in vivo, especially when using disk geometry scaffolds [78]. Despite these interesting results, biomechanics were not mentioned.

**Figure 11:**
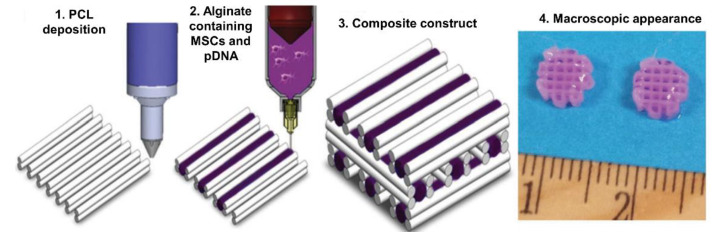
Schematic representation of the bioprinting process with co-printing of PCL and the bioprinting of the bio-ink composed of alginate, MSCs, and nHAP-pDNA complexes. Adapted from [79].

For osteochondral regeneration, HAP has been also used. For example, Cunniffe et al. [79] fabricated an RGD-γ-irradiated alginate and porcine bone marrow MSC bio-ink. Interestingly, they included nanohydroxyapatite complexed with plasmid DNA encoding TGF-β3 and BMP-2 growth factors. Extrusion-based bioprinting was used accompanied by PCL co-printing as a supporting mesh to provide mechanical stability to the construct (Figure 11). They achieved good cell viability with this co-printing technique, as well as high transfection rates. Moreover, higher ECM production and mineralization were observed with the plasmid-encoding growth factors. Finally, they performed an in vivo study by implanting the scaffold subcutaneously in nude mice. As a result, bone formation, immature osteoid, and vascularization were detected, suggesting a feasible approach for bone regeneration [79]. In a recent work by the same research group, Freeman et al. [80] applied this co-printing procedure with bio-ink extrusion and PCL to manufacture scaffolds. In this case, two different bio-inks were proposed: vascular and osteoinductive bio-inks. The vascular bio-ink was composed of RGD-γ-irradiated alginate/methylcellulose and hydroxyapatite nanoparticles loaded with the growth factor VEGF. On the other hand, the osteoinductive bio-ink was based on RGD-γ-irradiated alginate/methylcellulose/Laponite/BMP-2 and porcine bone marrow MSCs. The obtained scaffolds were implanted subcutaneously in nude mice. VEFG gradient scaffolds were bioprinted with the vascular bio-ink, showing vascularization in vivo. In contrast, after the implantation of the osteoinductive scaffolds, bone formation and sustained release of BMP-2 due to Laponite clay were observed. Interestingly, the scaffolds containing both bio-inks were fabricated and evaluated in rat femoral defects. Results showed an increase in vessel volume as well as in new bone formation, indicating a promising therapeutic approach for bone regeneration [80]. As a point for improvement, further studies on mechanical properties should be performed.

Another bioceramic that has been used for 3D bioprinting purposes is β-tricalcium phosphate (TCP). Like HAP, TCP has been reported to promote osteogenic differentiation of MSCs; therefore, Kim et al. [81] included this bioceramic in their collagen-type-I-based bio-ink. Highly porous scaffolds were obtained through extrusion-based bioprinting. However, the mechanical properties should have been improved, since the native bone values were not achieved. Biological evaluation was first carried out with preosteoblast cells (MC3T3-E1), showing good cell viability and proliferation as well as enhancement of mineralization after bioprinting. Then, human adipose-derived MSCs were used to evaluate their osteogenic differentiation capacity. As a result, TCP-containing scaffolds demonstrated matrix mineralization based on the increase in calcium and phosphorus. Furthermore, osteogenic markers and osteogenic gene expression increased with TCP. Interestingly, osteogenic differentiation was also shown in alginate/TCP/hMSCs scaffolds without the need for adding an osteogenic culture medium [81]. Consequently, the use of TCP could be a promising approach for the manufacture of scaffolds for bone regeneration. Nevertheless, low mechanical properties (5.94 MPa) make these scaffolds best considered as temporary substitutes for damaged bone.

Glass can also be incorporated in the fabrication of scaffolds for bone regeneration. In the work proposed by Kolan et al. [82], the authors included the highly angiogenic borate bioactive glass (13-93B3), which was approved by the FDA for the treatment of skin burns and chronic wounds. They developed a bio-ink composed of alginate/GelMA and human adipose-derived MSCs using extrusion-based bioprinting to biofabricate the scaffolds. Like in the previous works, they carried out a PCL co-printing procedure to improve the stability and mechanical properties (from 0.3 MPa to 50.6 MPa). Then, two approaches were proposed: one consisting of the addition of the glass to the PCL, and another consisting of the inclusion of the glass directly in the bio-ink. Results showed a decrease in cell viability within the days after bioprinting when the glass was included within the PCL. The authors argued that the solubility of the glass may have produced solutes that increased the pH, which was harmful to the cells. Furthermore, alginate/GelMA layers lost their stability over time, which accentuated the decrease in cell viability. On the other hand, when the glass was added directly to the bio-ink, an initial cell viability decrease was observed due to pH shock toxicity, but cell recovery was shown during the days after bioprinting, since the glass promoted crosslinking between the alginate and GelMA, making the scaffolds more stable. The authors concluded that the glass could be interesting to manufacture more stable scaffolds, but that dynamic culture systems should be implemented or glass concentration should be optimized in order to avoid toxicity [82].

**Figure 12:**
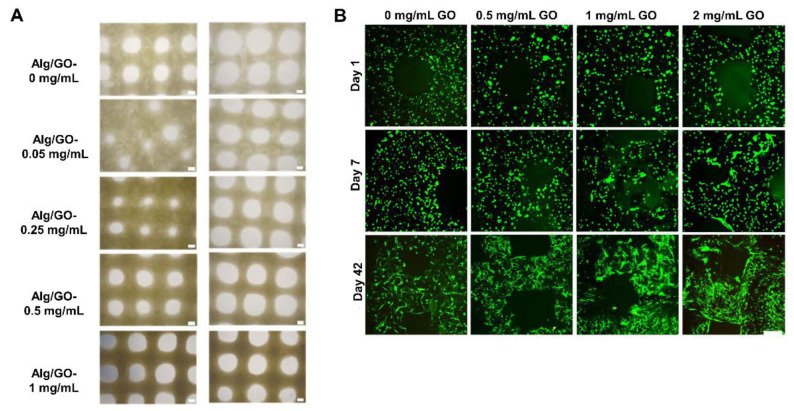
Graphene oxide scaffolds: (**A**) Optical images of the top view of the printed scaffolds, indicating better printability when GO increases from 0.05 mg/mL to 1 mg/mL. Scale bars = 300 μm. Adapted from [83]. (**B**) Cell viability in the 3D-bioprinted GO scaffolds at days 1, 7, and 42. Living cells are depicted in green, and dead cells are in red. Scale bar = 50 μm. Adapted from [84].

Another interesting approach for bone regeneration is the incorporation of inorganic components into the bio-ink. Among them, graphene oxide (GO) has gained notoriety because its functional groups enable the creation of strong interactions with various molecules [83,86]. Consequently, hydrogels with high mechanical properties have been obtained. Choe et al. [83] added GO into a sodium alginate and human MSC bio-ink. They showed that by increasing the GO concentration from 0.05 mg/mL to 1 mg/mL, the bio-ink’s rheological properties, printability (Figure 12A), scaffold stability, and mechanics increased. Interestingly, GO protects cells from oxidative stress, and promotes osteogenic differentiation in terms of alkaline phosphatase enzyme (ALP) production and mineralization, along with osteogenic gene expression [83]. Likewise, Zhang et al. [84] included different concentrations of GO in their bio-ink composed of sodium alginate, gelatin, and human bone marrow MSCs. The GO improved extrusion-based printability and scaffold fidelity. Furthermore, as Figure 12B shows, cell viability and proliferation were good despite increasing GO concentrations from 0.5 mg/mL to 2 mg/mL. Furthermore, osteogenic differentiation was shown in scaffolds containing GO. Importantly, higher GO concentrations improved DNA content as well as mineral volume after bioreactor culture [84]. These two studies showed that the use of GO could be an interesting option for bone bioprinting, because it would not only improve the physical properties of the scaffold, but also promote osteogenesis.

### 3.3. 3D Bioprinting in Osteochondral Units

The latest and most innovative approach to regenerate osteochondral injuries is the manufacture of 3D structures that contain both cartilage and bone tissues. Despite the fact that this is a complicated challenge due to all of the intrinsic characteristics that each tissue must meet, interesting and promising advances have been achieved (Table 4).

For example, in a recent study, Sun et al. [87] combined extrusion-based bioprinting with PCL printing. By depositing thicker layers on the bottom and thinner layers on the superficial part, a PCL gradient scaffold was fabricated in which a bio-ink composed of gelatin, fibrinogen, HA, glycerol, and rabbit bone marrow MSCs was deposited between the PCL layers. Importantly, PLGA microspheres loaded with growth factors (i.e., TGF-β3 for the superficial part, and BMP-4 for the deeper part) were added. Thus, the authors aimed to generate cartilage in the superficial part and bone in the deep part. The obtained scaffold showed excellent mechanical properties that were similar to those of native tissue. Good cell viability and proliferation were also achieved, together with a sustained growth factor release. Importantly, chondrogenic ECM production was observed in the superficial layers after the implantation in nude mice. Finally, the scaffold was implanted in the rabbits’ knees, and chondrogenic gene expression was quantified in the superficial layers, whereas osteogenic markers were found in the deeper layers [87]. These results seem to be promising for the regeneration of joint injuries; however, a further evaluation of joint functionality should be carried out.

**Figure 13:**
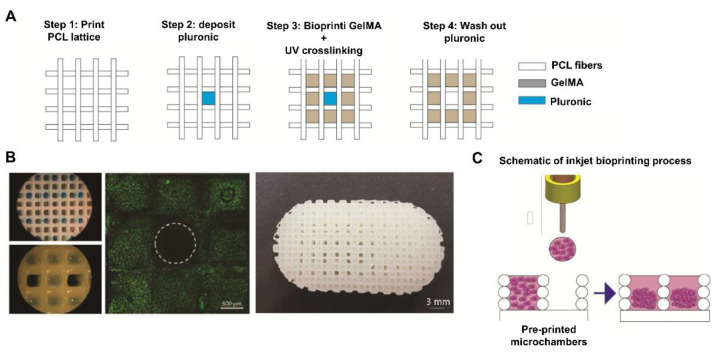
Multi-tool bioprinting procedure: (**A**) Schematic images of PCL printing and GelMA and pluronic bioprinting to create the bone region. (**B**) Macroscopic images of bioprinted scaffold. Live/dead analysis of MSC-laden GelMA bio-ink including microchannels after washing out pluronic. Scale bars = 0.5 mm and 3 mm. (**C**) Inkjet bioprinting procedure to obtain the cartilage part. Adapted from [88].

In another study, Daly et al. [88] developed a multi-tool bioprinting procedure to manufacture tibial-like curvature structures (Figure 13). First, by using the printing technique, PCL structures containing microchambers were fabricated. Then, a bio-ink containing GelMA and porcine bone marrow MSCs was deposited inside the microchambers by extrusion-based bioprinting to manufacture the bone part (Figure 13A). Likewise, bio-ink-free microchannels were created with the sacrificial ink pluronic as a nutrient diffusion system. Finally, inkjet bioprinting was performed using only the culture media with MSCs co-cultured with chondrocytes as a bio-ink (Figure 13C). Afterwards, the obtained scaffold was cultured in a bioreactor. The results showed high cell viability (Figure 13B) together with an osteochondral pathway in the bone part and cartilaginous ECM production in the cartilage part. Importantly, the GAG content and the mechanical properties were in the range of native tissue [88]. Recently, the same research group focused on how to bring these structures to an in vivo study. To do so, first, they had to devise a system for fixation to the articular bone. Thus, Burdis et al. [89] developed a biodegradable microwell array pin of PCL via printing technology in order to insert it in a hole that was created in the subchondral bone. Then, a porcine bone marrow MSC suspension was deposited on this device through inkjet bioprinting. The results showed excellent cell viability and quantification of cartilage ECM components. Importantly, after the culture of the scaffold in a bioreactor, cartilage-like assembly in terms of collagen alignment was observed [89]. Although this study managed to devise an interesting method for scaffold implantation in the joint, mechanical testing remains pending.

Another approach was the one proposed by Deng et al. [90] They developed two bio-inks: cartilage and bone bio-inks. Their cartilage bio-ink was composed of gelatin methacrylate, silk fibroin methacrylate, and rabbit chondrocytes. Moreover, parathyroid hormone (PTH), which was reported to inhibit chondrocyte hypertrophy, was added. On the other hand, the bone bio-ink was based on gelatin methacrylate, silk fibroin methacrylate, and rabbit bone marrow MSCs. Extrusion-based bioprinting was used to create the silk fibroin gradient scaffolds. As a result, good printability and cell viability after bioprinting were obtained. In addition, the hyaline cartilage phenotype was maintained due to PTH. Interestingly, the scaffolds were implanted in rabbit articular osteochondral defects, showing good regeneration in vivo. In contrast, although the mechanical properties increased in the bone zone (211.10 kPa), they did not resemble those of native bone [90]. In another work, Kilian et al. [91] used alginate methylcellulose (Alg-MC) with human chondrocytes (hC) to fabricate the cartilage part, followed by an Alg-MC-hC mixed with calcium phosphate cement (CPC) to create calcified cartilage, and CMC alone to fabricate the bone part. They performed the bioprinting via extrusion. The results showed a decrease in viability with the CPC layer, even though chondrogenic markers such as GAGs and collagen type II were present [91].

## 4. Current Limitations of 3D Bioprinting

Despite these advances, one of the greatest difficulties is the fabrication of scaffolds that possess similar mechanical properties to those of native tissues. Therefore, the inclusion of other novel polymers such as silk fibroin has been studied, resulting in an interesting component for these purposes. As an alternative, chemical modification, such as polymer methacrylation, has also been applied. However, this is a goal that has not been fully achieved yet—especially in the case of bone bioprinting, which requires superior mechanical properties to cartilage tissue. Additionally, printing technology has been combined with bioprinting in order to produce synthetic polymers with good mechanical properties, such as PCL, with more acceptable biomechanical values. On the other hand, in the case of bone, bioceramics such as HAP or inorganic components such as GO have been included in scaffolds to achieve desirable biomechanics. Furthermore, osteoconductivity and osteoinduction have also been enhanced by adding these components.

Another challenge is the fabrication of the entire osteochondral unit. Lately, thanks to the advantages provided by 3D bioprinting techniques, some interesting results have been obtained. Among these, the manufacture of biomaterial gradient scaffolds together with the addition of specific growth factors to the bio-inks has been shown to be successful.

From a more general point of view, while 3D printing has been acquiring clinical importance, 3D bioprinting technology is in its beginnings, and there has been no translation to clinical practice yet. This lack of translation could be due to safety, ethical, and regulatory issues. Safety problems are related to the materials used to fabricate the bio-inks. For instance, the use of mesenchymal and pluripotent stem cells has been widely expanded when it comes to manufacturing bio-inks, but they are not exempt from problems such as tumor formation [92]. Moreover, cell behavior may change after exposing the cells to high bioprinting pressures or to crosslinkers such as UV light [93]. Likewise, biomaterials of non-human origin—such as alginate or gelatin—are widely utilized in the bioprinting field, but few studies have focused on the immunological response in vivo or on possible pathogen transmission. In addition, treatments of animal origin could be ethically controversial to apply to certain populations with religious and cultural beliefs. On the other hand, the high cost of the bioprinting process could lead such therapy to be accessible only to people with high purchasing power, which would be ethically questionable [92]. Finally, there is a discussion among regulatory agencies about the category in which the bioprinted scaffolds should be classified. In fact, a bioprinting scaffold can be a medical device, an advanced therapy medicinal product, and a medicinal product at the same time, with different regulatory requirements and protocols [93].

## 5. Conclusions

The rise of 3D bioprinting technology has brought a wide range of opportunities to the tissue engineering field. Currently, 3D bioprinting technology allows the fabrication of structures that can regenerate tissues such as cartilage and bone. Thus, it opens the door to achieving the treatment of certain osteoarticular diseases, such as OA, which not only has a high prevalence, but also entails an economic burden on healthcare systems. Until the appearance of this technology, current scaffolding techniques have failed in producing osteochondral tissue substitutes with adequate mechanical properties and a multilayered internal structure. However, 3D bioprinting allows the layer-by-layer manufacturing of structures that can resemble native tissues. Moreover, this technique can use a wide range of biomaterials, among which natural polymers stand out. Gelatin and alginate, together with the ECM components, such as collagen, HA, and chondroitin sulfate, have shown to be promising for osteochondral regeneration, since they demonstrate good biocompatibility, biodegradability, and non-cytotoxic properties. On the other hand, synthetic components such as methacrylate polymers and PCL, along with ceramics and graphene oxide, have also been applied for scaffold fabrication—especially in bone regeneration, since it requires high mechanical properties, osteoconduction, and osteoinduction. In addition, 3D bioprinting allows the use of high cell densities. Among them, chondrocytes have mostly been used to evaluate the biocompatibility of the ink and the bioprinting process. Meanwhile, the use of MSCs that have the ability to differentiate into specific cell types has attracted attention for scaffolds that regenerate cartilage and bone.

Other advantages of 3D bioprinting are that it is fast, automatic, and reproducible. Furthermore, this technique may bring personalized medicine closer to clinical practice since, on the one hand, biomaterials may be improved for specific organs and patients, and on the other hand, the doses of molecules, drugs, or biological components may be adjusted for each patient. For all of these reasons, 3D bioprinting may be a feasible technology to manufacture 3D structures that regenerate cartilage, bone, and both tissues at the same time. However, the improvement of biomechanical properties and the biofabrication of multilayered scaffolds to simulate native tissues are still drawbacks that need to be addressed. Additionally, safety, ethical, and regulatory concerns should also be taken into consideration in the future.

## Figures and Tables

**Table 1:** Summary of the benefits and side effects of the current treatments for OA.

Treatment	Positive Effects	Side Effects
Topical treatment	- Effective and easy to administrate - Generally well-tolerated	- Ineffective in advanced stages of OA
Acetaminophen or paracetamol	- First choice treatment - Good relieving pain	- Controversy about the long-term effectiveness - No anti-inflammatory effects - Hepatotoxic when abused
Oral NSAIDs	- First-choice treatment - Good for relieving pain and improving joint function	- Gastrointestinal and cardiac issues in the long-term and when abused
SYSADOA	- Safe and well-tolerated - Pain relief and improvement in joints’ physical function	- Unclear therapeutic mechanisms - Discrepancies among therapeutic guides
Intra-articular injectable HA	- Safe and well-tolerated - Anti-inflammatory effects and pain reduction	- Benefits only in the short-term period - Repetitive intra-articular injections - Only useful in mild and moderate stages of OA
Intra-articular injectable corticosteroids	- Good for reducing joint inflammation and dysfunction	- Benefits only in the short-term period - Repetitive intra-articular injections - Controversial benefits in knee joints and in the long-term
Opiates	- Excellent painkillers when other treatments fail	- Tolerance and dependence - Negative benefit/risk ratio - Highly discouraged
Surgery	- Last therapeutic option - Relevant improvement, especially in young patients	- More likely to have complications associated with surgery in the elderly population - Probability of rejection - Pain is still recurrent
PRP	- Relief of OA symptoms - No side effects	- Limited to knees - Variability among patients - Unclear dosage and plasma extraction protocols - Efficacy decreases with NSAIDs
Spherox™	- Osteochondral regeneration - General improvement	- Not available in all hospitals - High costs and long regulatory procedures - Contraindicated in advanced OA - Only applicable for knee defects

Acronyms—OA: osteoarthritis; NSAIDs: nonsteroidal anti-inflammatory drugs; SYSADOA: symptomatic slow-acting drugs in osteoarthritis; HA: hyaluronic acid; PRP: platelet-rich plasma.

**Table 2:** Summary of the 3D bioprinting studies for cartilage.

Bio-Ink	Cells	Technique	In Vivo	Results	Ref.
Type I COL	Rat chondrocytes	Extrusion-based bioprinting	Wistar Rats	Good printability Type II COL and GAG accumulation in vivo	[57]
ALG/COL ALG/agarose	Rat primary chondrocytes	Extrusion-based bioprinting	No	COL improves scaffold mechanical properties COL enhances cell viability after bioprinting Cells inside the collagen scaffold increase chondrogenic gene expression and GAG production	[58]
NFC/ALG NFC/ALG	HDiPSCs co-cultured with irradiated human chondrocytes	Extrusion-based bioprinting	No	NFC/ALG scaffolds show better results in terms of cells’ proliferation, pluripotency maintenance, and chondrogenic phenotype expression	[59]
ALG/pig dECM/TGF-β3	Human BMSCs	Extrusion-based bioprinting	No	High cell viability after bioprinting Sustained release of TGF-β3 from the scaffold Higher concentrations of ECM enhance cells’ chondrogenic differentiation, but also osteochondral differentiation in the long-term Native mechanical properties after the reinforcement with PCL fibers through 3D printing	[60]
Pig cartilage derived dECM/Gel/HA/glycerol/DMEM	Rabbit chondrocytes	Extrusion-based bioprinting	No	Scaffold mechanical properties increased by the addition of dECM Cell viability and proliferation are proportional to the dECM concentration in the scaffold dECM promotes cells to produce GAGs and COL.	[61]
SF/goat cartilage derived dECM/TGF-β3/PEG 400	Rabbit BMSCs	Extrusion-based bioprinting	Nude mice	Good printability dECM enhances cell proliferation, viability, and chondrogenic differentiation after bioprinting Sustained release of TGF-β3, which promotes GAG and COL production Cartilage ECM production as well as the increase in mechanical properties after in vivo implantation	[62]
SF/rabbit PRP/PEG 400	Rabbit chondrocytes	Extrusion-based bioprinting	No	PRP increases scaffold mechanical properties Sustained release of growth factors that are found in the PRP from the scaffold PRP enhances cell viability and proliferation, and promotes cell chondrogenic differentiation after bioprinting	[63]
SF/Gel	Porcine primary chondrocytes	Extrusion-based bioprinting	Swiss inbred mice	Porous and printable scaffolds Low mechanical properties similar to those ofsoft tissues High cell viability and proliferation after bioprinting Cell chondrogenic differentiation inside scaffolds after bioprinting Implanted scaffolds do not provoke a long immune response	[64]
Hydroxybutyl CH/oxidized CS	Human ADMSCs	Extrusion-based bioprinting	C57BL/6 mice	Good biocompatibility in vivo Low immunotoxicity; decrease in cytokines that degrade cartilage	[65]
Norbornene-modified HA	Bovine BMSC	In situ crosslinkable extrusion-based bioprinting	No	In situ crosslinking technique with visible light exposure High cell viability after bioprinting with this technique Cells’ chondrogenic differentiation and scaffold mechanical properties increase after bioprinting	[66]
HAMA/GelMA	Sheep ADMSCs	In situ handheld extrusion-based bioprinting “Biopen”	Chondral defect sheep	Good handling and applicability of Biopen Cartilage regeneration and mechanical properties in vivo are good with Biopen, but no differences compared to conventionally bioprinted scaffolds Lack of adhesion to host tissue	[67]
GelMA/PCL	Sheep chondrocytes	Extrusion-based bioprinting + PCL 3D printing	No	High cell viability and proliferation after bioprinting Good chondrogenic functionality of cells after bioprinting High mechanical properties after the addition of PCL	[68]
GelMA/PCL	Equine MSCs	Extrusion-based bioprinting + PCL melt electrowriting	No	High cell viability and proliferation after bioprinting using both techniques Cells produce GAGs and COL after bioprinting using both techniques	[69]
GelMa/PEGDA/TGF-β1-PLGA nanospheres	Human BMSCs	Stereolithography-based 3D bioprinting	No	PEGDA improves mechanics and printability High cell viability and proliferation after bioprinting Sustained release of TGF-β1 from the scaffold, which promotes cells’ chondrogenic differentiation after bioprinting	[70]
ALG	Human ADMSCS	Extrusion-based bioprinting + aspiration-assisted bioprinting	No	Layered scaffold simulating the deep and superficial layers of native cartilage High cell viability after bioprinting Mechanical properties similar to those of native cartilage Cells deposit COL fibers aligned with designed orientation	[71]
GelMA/GG GelMA/GG/(HAMA)	Equine chondrocytes/MSCs/ACPCs	Extrusion-based bioprinting	No	HAMA improves printability No differentiated cells show better results in terms of cartilage ECM production and differentiation Layered scaffold with HAMA bio-ink simulating a superficial cartilage layer with ACPCs and a middle/deep layer with MSCs Cells in layered scaffolds show good chondrogenic differentiation, but no differences between layers	[72]

Acronyms—COL: collagen; GAGs: glycosaminoglycans; ALG: alginate; NFC: nanofibrillated cellulose; HDiPSCs: human-derived induced pluripotent stem cells; HA: hyaluronic acid; dECM: decellularized extracellular matrix; BMSCs: bone marrow stem cells; SF: silk fibroin; PEG: polyethylene glycol; PRP: platelet-rich plasma; Gel: gelatin; CH: chitosan; CS: chondroitin sulfate; ADMSCs: adipose-derived mesenchymal stem cells; GelMA: gelatin methacrylate; HAMA: hyaluronic methacrylate; PCL: polycaprolactone; PEGDA: polyethylene glycol diacrylate; GG: gellan gum; PLGA: poly-lactic-co-glycolic acid; ACPCs: articular cartilage progenitor cells.

**Table 3:** Summary of the 3D bioprinting studies for bone regeneration.

Bio-ink	Cells	Technique	In Vivo	Results	Ref.
ALG/PVA/HAP	Murine calvaria 3T3-E1 cells	Extrusion-based bioprinting	No	PVA/HAP increase bio-ink rheological properties Good cell viability after printing Low mechanical properties	[77]
Nano-HAP/type I COL	Murine D1-MSCs	Laser-based bioprinting	Calvaria defect rats	Manufacture of scaffolds with two geometries: ring and disk High viability and proliferation after bioprinting Bone regeneration in vivo using disk scaffolds	[78]
RGD-γ-irradiated ALG/nano-HAP pDNA complexes encoding TGF-β3 and BMP-2 growth factors	Porcine BMSCs	Extrusion-based bioprinting + PCL 3D printing	Nude mice	High cell viability using PCL co-printing technique High transfection rates Bone ECM production and mineralization Bone formation, immature osteoid detection, and vascularization in vivo	[79]
Vascular bio-ink: RGD-γ-irradiated ALG/MC/nano-HAP nanoparticles loaded with VEGF Osteoinductive bio-ink: RGD-γ-irradiated ALG/MC/LAP/BMP-2	Porcine BMSCs	Extrusion-based bioprinting + PCL 3D printing	Nude mice and femoral-defect rats	Increased vascularization in nude mice with VEGF gradient scaffolds Bone formation and BMP-2 sustained release with osteoinductive scaffolds in nude mice Increase in vessel volume and new bone formation using both bio-ink-based scaffolds in femoral-defect rats	[80]
Type I COL/TCP	Preosteoblast cells (MC3T3-E1) And human ADMSCs	Extrusion-based bioprinting	No	Highly porous scaffolds Good cell viability and proliferation after bioprinting TCP enhances scaffold mineralization after bioprinting with preosteoblast cells TCP promotes osteogenic markers and gene expression in hADMSCs after bioprinting	[81]
ALG/GelMA/highly angiogenic borate bioactive glass (13-93B3)	Human ADMSCs	Extrusion-based bioprinting + PCL 3D printing	No	Glass enhances scaffold stability after bioprinting by promoting alginate–GelMA crosslinking Glass solutes induces a pH increase in the media that is toxic to cells	[82]
ALG/GO	Human MSCs	Extrusion-based bioprinting	No	GO enhances bio-ink’s rheological properties Printability and scaffold mechanics are improved by GO GO protects cells from oxidative stress and promotes their differentiation to bone	[83]
ALG/Gel/GO	Human BMSCs	Extrusion-based bioprinting	No	GO increases printability and scaffold fidelity Good cell viability, proliferation, and osteogenic differentiation after bioprinting Higher GO concentrations increase DNA content and mineralization	[84]

Acronyms—ALG: alginate; PVA: polyvinyl alcohol; COL: collagen; HAP: hydroxyapatite; BMSCs: bone marrow stem cells; PCL: polycaprolactone; ECM: extracellular matrix; MC: methylcellulose; LAP: Laponite; TCP: β-tricalcium phosphate; ADMSCs: adipose-derived mesenchymal stem cells; GelMA: gelatin methacrylate; GO: graphene oxide; Gel: gelatin.

**Table 4:** Summary of the 3D bioprinting studies for cartilage and bone together.

Bio-ink	Cells	Technique	In Vivo	Results	Ref.
Gel/FGN/HA/glycerol and PLGA microspheres loaded with TGF-β3 for superficial layers and BMP-4 for deeper layers.	Rabbit BMSC	Extrusion-based bioprinting + PCL 3D printing	Nude mice; rabbit knee defects	PCL gradient scaffolds to give structure Excellent mechanical properties Good cell viability and proliferation after bioprinting Sustained release of growth factors Cartilaginous ECM production in mice after subcutaneous implantation Chromogenic gene expression in the superficial layer and detection of osteogenic markers in the deeper layers in vivo in rabbits	[87]
GelMA/pluronic	Porcine BMSCs co cultured with chondrocytes	Inkjet-based bioprinting + extrusion-based bioprinting + PCL 3D printing	No	Good integration of three techniques Observation of osteochondral and chondral pathways GAG contents and mechanical properties comparable to those in native tissue	[88]
Bio-ink free	Porcine BMSCs	Inkjet-based bioprinting + PCL 3D printing	No	Excellent cell viability after bioprinting Cartilage-like ECM production COL alignment similar to that of native tissue after dynamic culture.	[89]
Cartilage bio-ink: GelMA/SFMA/PTH Bone bio-ink: GelMA/SFMA	Rabbit chondrocytes Rabbit BMSCs	Extrusion-based bioprinting	Articular osteochondral defect rabbits	Mechanical gradient scaffold PTH inhibits chondrocyte hypertrophy, maintaining the hyaline phenotype Osteochondral regeneration in vivo	[90]
ALG/MC/CPC	Human chondrocytes	Extrusion-based bioprinting	No	Creation of three zone scaffolds Cell viability decrease with CPC Chondrogenic presence	[91]

Acronyms. Gel: gelatin; FGN: fibrinogen; HA: hyaluronic acid; PLGA: poly-lactic-co-glycolic acid; PCL: polycaprolactone; BMSCs: bone marrow stem cells; ECM: extracellular matrix; GAGs: glycosaminoglycans; COL: collagen; GelMA: gelatin methacrylate SFMA: silk fibroin methacrylate; PTH: parathyroid hormone; ALG: alginate; MC: methylcellulose; CPC: calcium phosphate cement.
